# Identification of genetic mutations conferring tedizolid resistance in MRSA mutants

**DOI:** 10.1007/s10096-025-05157-x

**Published:** 2025-05-13

**Authors:** Nesma B. Goda, Amira M. El-Ganiny, Tharwat R. El-khamissy, Fares Z. Najar, Ashraf A. Kadry

**Affiliations:** 1 Microbiology and Immunology Department, Faculty of Pharmacy, Egyptian Russian University, Badr, Egypt; 2 Microbiology and Immunology Department, Faculty of Pharmacy, Zagazig University, Zagazig, 44519 Egypt; 3 High-Performance Computing Center MS #105, Oklahoma State University, Stillwater, OK 74078 USA

**Keywords:** Tedizolid, Cross-resistance, Mutation, MRSA, *mepB*, *mepRAB* operon

## Abstract

**Purpose:**

In light of previous studies eliminating the involvement of gene-mediated mechanisms in developing tedizolid resistance, our study elucidates the ability of mutation-mediated mechanisms to confer oxazolidinones cross-resistance in methicillin-resistant *Staphylococcus aureus* (MRSA). With further investigation of the identified mutations and their relation to tedizolid resistance. Additionally, the involvement of *rpoB* mutations in acquiring resistance to tedizolid was also investigated.

**Methods:**

Five *cfr-*negative, methicillin-resistant *Staphylococcus aureus* clinical isolates were subjected to in vitro selection to develop linezolid-resistant mutants. The resultant mutants were tested for acquiring tedizolid cross-resistance, whole genome sequencing was performed twice, followed by variant calling and annotation. Detected mutations were analyzed for their relatedness to the developed resistance.

**Results:**

Mutations considered relevant to tedizolid resistance were detected in *rpoB* gene encoding β-subunit of the RNA polymerase enzyme and *rplC* gene encoding the 50S ribosomal protein L3. Additionally, mutations in *mepB* gene, part of the *mepRAB* operon were detected and believed to contribute to acquiring linezolid resistance.

**Conclusion:**

To the best of our knowledge, our findings are the first to report the 50S ribosomal protein L3 mutation Gly152Asp to solely confer cross-resistance to both linezolid and tedizolid oxazolidinones. In addition, we report the emergence of cross-resistance between oxazolidinone antibiotics and rifampin through a single amino-acid substitution occurring within the Rifampin Resistance Determining Region (RRDR). Furthermore, *mepB* mutations reported in our results support a theory implying a second MepR-independent mechanism regulating the *mepRAB* operon, and are believed to be responsible for the acquired linezolid resistance in our study.

**Supplementary Information:**

The online version contains supplementary material available at 10.1007/s10096-025-05157-x.

## Introduction

*Staphylococcus aureus* is a devastating Gram-positive pathogen that can cause a variety of infections, ranging from superficial skin infections to endocarditis. Having the ability to resist the action of the major class of beta-lactams, methicillin-resistant *Staphylococcus aureus* has been a major concern for health care professionals due to the limitations in treatment options. Currently, treatment options for MRSA infections include vancomycin and newer glycopeptides, clindamycin, and the oxazolidinone drugs linezolid and tedizolid [1]. 

Linezolid; the first member of oxazolidinone antibiotics was approved by the FDA for clinical use in early 2000 [2]. This was followed by the rapid emergence of linezolid resistance in *Staphylococcus aureus* clinical isolates, which has been reported in several studies [3, 4]. Resistance mechanisms to linezolid are many, varying between gene-mediated and mutation-mediated mechanisms [5–7].

Regardless of the reason behind the resistance, efforts needed to be made to come up with an effective alternative. Which led to the development of tedizolid. Although both linezolid and tedizolid share the same mechanism of inhibiting bacterial protein synthesis by binding to the Peptidyl Transferase Center (PTC) of the 50S ribosomal subunit [8], tedizolid’s chemical structure was modified to overcome the heavily reported *cfr*-mediated linezolid resistance mechanism. Thus, providing a higher affinity to its binding site at the PTC, resulting in a greater efficacy against *cfr*-bearing linezolid resistant strains [9]. Studies testing tedizolid’s efficacy against methicillin-linezolid resistant *S. aureus* strains have concluded that indeed tedizolid resistance is not mediated through the *cfr* gene, but rather is due to a mutation-mediated mechanism [10, 11].

Our study aims to investigate whether a mutation-mediated mechanism could confer cross-resistance to linezolid and tedizolid oxazolidinones. Furthermore, the role of *rpoB* gene in developing tedizolid resistance was also investigated.

## Materials and Methods

### Isolates selection and identification

Five *S. aureus* clinical isolates were selected from the culture collection of the Microbiology & Immunology Department at Faculty of Pharmacy, Zagazig University. Isolates selection was determined based on their resistance to methicillin and susceptibility to linezolid. Initial identification was achieved by growing on Mannitol Salt Agar (MSA) plates and the appearance of golden-yellow colonies on nutrient agar plates, followed by a tube coagulase test to confirm identity as *S. aureus*. Molecular identification involved taxonomic classification of the obtained Whole Genome Sequencing (WGS) data, which also confirmed identity as *S. aureus*.

### Antimicrobial susceptibility testing: disc diffusion method

Using cefoxitin discs, disc diffusion protocol was followed to screen for methicillin resistance [12]. Antibiotic resistance profile for each isolate was also determined for agents belonging to PhLOPSa antibiotics (Phenicols, Lincosamides, Oxazolidinones, Pleuromutilins, and StreptograminA). In addition, susceptibility to rifampin was tested due to its relevance to *rpoB* mutations. *S. aureus* ATCC 25923 was included as a routine quality control measure.

### Antimicrobial susceptibility testing: broth microdilution method

Minimum Inhibitory Concentrations (MIC) for linezolid and tedizolid at baseline were determined for each isolate. Linezolid (Global Napi Pharmaceuticals, Egypt) was purchased, 128 ug/ml working solution was prepared and two-fold serial dilution was performed so that the last microtiter plate well contained an antibiotic concentration of 0.5 ug/ml.

Tedizolid (CAS856866-72-3) was purchased (Thermo Fisher Scientific, USA) and a concentration of 100 ug/ml was used as a working solution. Two-fold serial dilution was performed so that the last microtiter plate well contained an antibiotic concentration of 0.09 ug/ml. CLSI protocol for broth microdilution method was followed [13].

### Detection of *mecA* Gene by Polymerase Chain Reaction

All five isolates were analyzed for acquisition of *mecA* gene responsible for methicillin resistance. The following primer sequences were used, ***mecA*-fw**: 5′ GTA GAA ATG ACT GAA CGT CCG ATA A 3′ and ***mecA*-rv**: 5′ CCA ATT CCA CAT TGT TCG GTC TAA 3′ [14]. ATCC 43300 was also amplified for use as a positive control. For a single 25 ul PCR reaction the following were added: 12.5 ul of One PCR Master Mix (GeneDireX, USA), 1.25 ul of each primer, 2 ul crude DNA extract, and 8 ul nuclease free water. Cycling conditions were as follows: Initial denaturation at 94 °C for 3 min, followed by 30 cycles of Denaturation: 30 s at 94 °C, Annealing: 1 min at 52 °C, Extension: 1 min at 72 °C and final extension at 72 °C for 5 min. PCR products were visualized on 1.5% agarose gel using Ethidium Bromide (EtBr) dye in 1X Tris-acetate-EDTA buffer (TAE).

### Detection of *cfr* Gene by Polymerase Chain Reaction

Once again, the five isolates were tested for the possession of *cfr* gene responsible for linezolid resistance and PhLOPSa phenomenon. The following primer sequences were used, ***cfr*-fw**: 5′ TGA AGT ATA AAG CAG GTT GGG AGT CA 3′ and ***cfr*-rv**: 5′ ACC ATA TAA TTG ACC ACA AGC AGC 3′ [15]. Cycling reaction and conditions were as those followed for *mecA* gene detection with the only difference being the annealing temperature. Different annealing temperatures were used ranging from 48 °Cto 57 °C to confirm the negative acquisition of the gene. Visualization was done using 1% agarose gel and EtBr dye in 1X TAE.

For the polymerase chain reaction, a crude extract of DNA was obtained using boiling method protocol [16] with minor modifications. Briefly, three colonies were suspended in 50 ul pyrogen free water followed by heating at 99 °C for 10 min and icing for 1 min. After centrifugation at 10,000 rpm for 1 min, the supernatant was drawn by pipetting and 2 ul of each sample was used as a template.

### In vitro selection of linezolid resistant mutants

Initially linezolid susceptible isolates **(**Table 1**)** were subjected to in vitro selection to develop linezolid resistance following the protocol mentioned by Shen et al. [17]. Following this protocol over 3 intermittent months enabled us to successfully grow MRSA resistant mutants at linezolid concentrations of 16 ug/ml. After establishing linezolid resistance, these mutants were tested again against tedizolid to check whether cross-resistance had developed, which would be reflected by an increase in MIC values **(**Table 2**)**.

### Whole genome sequencing

In order to investigate the mechanisms of acquiring tedizolid resistance, each isolate was subjected to WGS twice, once before and again after developing linezolid resistance. This dual sequencing approach was followed for the purpose of excluding mutations that may have been naturally present in tested isolates.

Following the manufacturer’s instructions, QIAamp^®^ DNA Minikit (QIAGEN, Germany) was used to extract genomic DNA. Followed by library preparation using the Nextera XT DNA library preparation kit (Illumina, USA). Sequencing was performed using Illumina MiSeqDx platform (Children’s Cancer Hospital 57357, Egypt). Sequence data generated from this study are available through the NCBI Sequence Read Archive (SRA) (PRJNA1173977).

### Bioinformatics analysis

Generated paired-end sequence reads for each isolate went through a series of steps summarized as follows: quality assessment and trimming of low quality reads and adaptor content using fastqc tool and trimmomatic. After trimming, reads were mapped to the *Staphylococcus aureus* N315 genome (NCBI RefSeq assembly GCF_000009645.1) using BWA mem. Followed by the removal of PCR duplicates using Picard.

*Staphylococcus aureus* N315 was found suitable due to its well characterization and clinical relevance. Variant calling and filtering were done using bcftools mpileup, bcftools call, and bcftools filter. The following filters were used: [QUAL > = 50 && FORMAT/DP > 10 && MQ > = 30]. Identified variants were annotated with SnpEff tool using the specific database for *Staphylococcus aureus* N315.

## Results

### Antimicrobial susceptibility testing

Cefoxitin was tested for MRSA identification, while chloramphenicol, clindamycin, and linezolid were chosen as they belong to PhLOPSa antibiotics. In addition, rifampin was tested due to its relevance to *rpoB* mutations. The results of disc diffusion method showed that all *S. aureus* isolates were resistant to cefoxitin, 4 out of the 5 isolates were resistant to both chloramphenicol and clindamycin, only one isolate was resistant to rifampin, while all the isolates were susceptible to linezolid (Table 1). The results of broth microdilution method indicated that all isolates were susceptible to linezolid, with 4 tedizolid susceptible and 1 intermediate isolate (Table 1).

**Table 1:** Antibiotic resistance profile for the five tested isolates at baseline

Clinical Isolate Number	Disc Diffusion Method					Broth Microdilution Method (ug/ml)	
	Cefoxitin	Chloramphenicol	Clindamycin	Linezolid	Rifampin	Linezolid	Tedizolid
1	Resistant	Resistant	Resistant	Susceptible	Susceptible	2	0.39
2	Resistant	Susceptible	Resistant	Susceptible	Resistant	2	0.39
3	Resistant	Resistant	Susceptible	Susceptible	Susceptible	4	1.56
4	Resistant	Resistant	Resistant	Susceptible	Susceptible	2	0.78
5	Resistant	Resistant	Resistant	Susceptible	Susceptible	2	0.78

tedizolid susceptible: ≤ 0.5 ug/ml, tedizolid intermediate: 1 ug/ml, tedizolid resistant: ≥ 2 ug/ml

linezolid susceptible: ≤ 4 ug/ml, linezolid resistant: ≥ 8 ug/ml

### Polymerase chain reaction

**Fig. 1:**
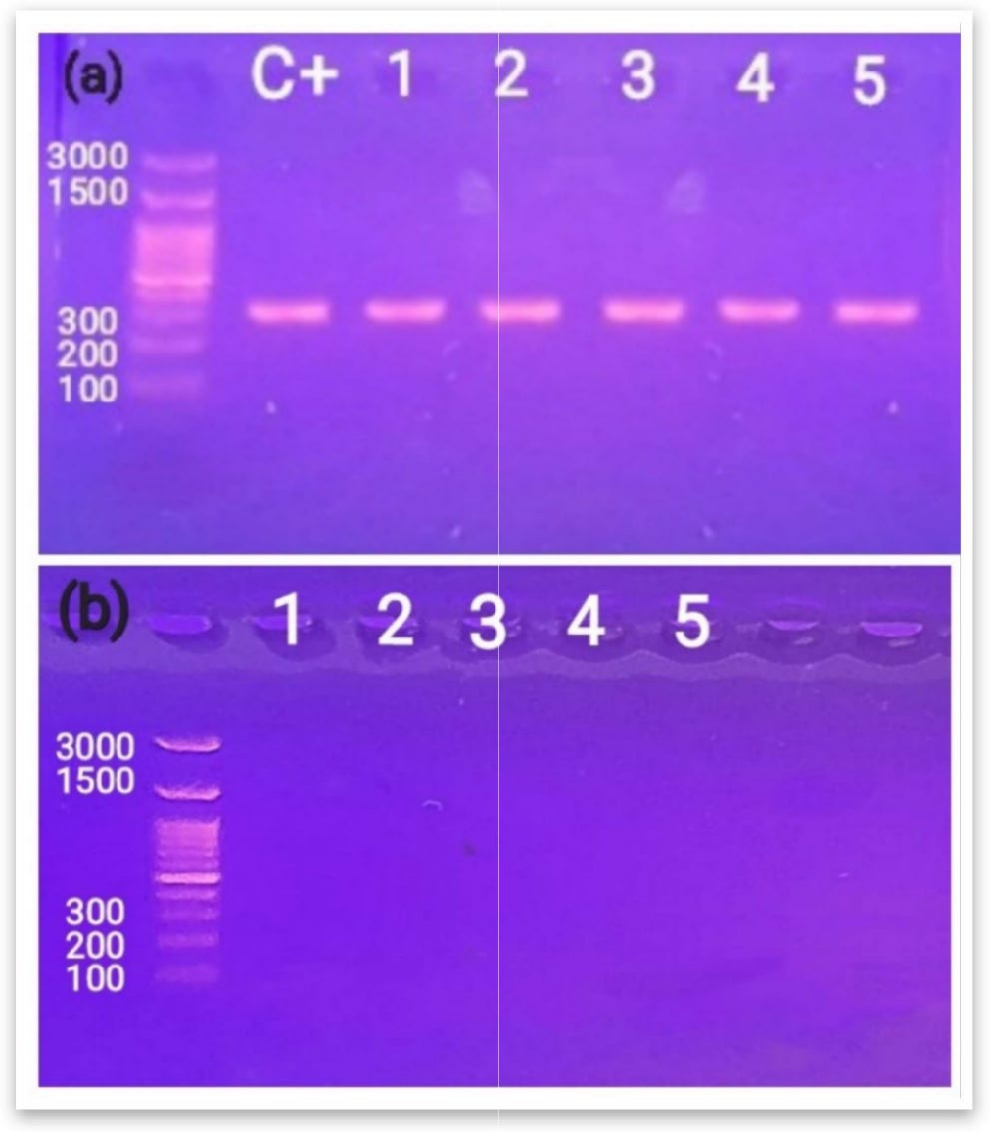
Electrophoretic graph of PCR products. (**a**) Detection of *mecA* gene; positive for all five isolates giving bands of 310 bp. ATCC 43300 was included as a positive control. (**b**) Detection of *cfr* gene; negative for all five isolates, not giving bands of 746 bp

PCR was performed for *mecA* gene detection in the five *S. aureus* isolates. The results confirmed that all isolates are MRSA giving a single band of 310 bp indicating the possession of *mecA* gene **(**Fig. 1a**)**. Additionally, PCR was used to investigate the presence of *cfr* gene, all tested isolates were *cfr*-negative as indicated in Fig. 1b.

### Induction of cross-resistance

Using in vitro selection protocol, MRSA mutants were obtained with the ability to grow at linezolid concentrations of 16 ug/ml. This was accompanied by a 4X increase in tedizolid MIC in isolates no. 1, 3, 4, and 5. While isolate no. 2 maintained its MIC at 0.39 ug/ml **(**Table 2**)**.

**Table 2:** Minimum inhibitory concentration of tedizolid against the five tested isolates before and after developing linezolid resistance

Isolate NO.	Tedizolid MIC before developing linezolid resistance (ug/ml)	Tedizolid MIC after developing linezolid resistance (ug/ml)
1	0.39	1.56
2	0.39	0.39
3	1.56	6.25
4	0.78	3.125
5	0.78	3.125

tedizolid susceptible: ≤ 0.5 ug/ml, tedizolid intermediate: 1 ug/ml, tedizolid resistant: ≥ 2 ug/ml

### Bioinformatics analysis

Three mutation-mediated mechanisms were identified by WGS and proposed to be involved in the acquired resistance (Table 3). Isolate no. 1 showed a single missense variant occurring in *rpoB* gene at position 1586C > T, making for the amino-acid substitution Ser529Leu. Isolate no. 5 also showed a single missense variant occurring in *rplC* gene at position 455C > T, making for the amino-acid substitution Gly152Asp. While isolate no. 2 showed two missense variants in *mepB* gene at positions 5A > G and 28T > A, making for the amino-acid substitutions Tyr2Cys and Tyr10Asn, respectively. Detected variants were visualized using the Integrative Genomics Viewer (IGV) tool as shown in online resource 1. Isolates no. 3 & 4 are not included in this table as a mechanism that may explain the acquired resistance was not concluded.

In addition to the detected mutations, WGS results also revealed the acquisition of the Antimicrobial Resistance (AMR) genes: *ermA* in isolates no. 1, 2, 4, and 5. *fexA* in isolates no. 3, 4, and 5. *catA* in isolate no. 1. WGS analysis also confirmed the negative acquisition of *cfr* gene in all tested isolates.

**Table 3:** A list of mutations identified following the induction of resistance

Conferred resistance	Affected gene	Isolate no.	Position in gene	Variant	Amino acid change	Impact
Mutation(s) conferring linezolid & tedizolid cross-resistance	*rpoB*	**1**	1586/3552	C > T	Ser529Leu	missense variant
	*rplC*	**5**	455/663	C > T	Gly152Asp	missense variant
Mutation(s) conferring linezolid resistance only	*mepB*	**2**	5/459 28/459	A > G T > A	Tyr2Cys Tyr10Asn	missense variants

## Discussion

Our study included five methicillin-resistant *Staphylococcus aureus* isolates, resistance to methicillin was determined using cefoxitin discs and confirmed by the detection of *mecA* gene. In addition to cefoxitin, resistance to chloramphenicol, clindamycin, and linezolid was also tested **(**Table 1**)** as they belong to PhLOPSa antibiotics whose resistance could be mediated by *cfr* gene [6]. Resistance to rifampin was also checked **(**Table 1**)** due to its relation to *rpoB* mutations which were proposed to be involved in tedizolid resistance [17].

All isolates showed linezolid susceptibility by both disc diffusion and broth microdilution methods **(**Table 1**)**, which comes consistent with PCR **(**Fig. 1b**)** and WGS results that confirmed negative acquisition of *cfr* gene in all five isolates.

Four isolates showed resistance to clindamycin **(**Table 1**)** which was later explained by whole genome sequencing analysis that revealed the possession of *ermA* gene in these isolates. The *ermA* gene mediates resistance to macrolides, lincosamides, and streptogramin B (MLS_B_) [18]. Chloramphenicol resistance was detected in four of the five isolates **(**Table 1**)**, which was explained by the possession of either *catA* gene responsible for chloramphenicol acetylation or *fexA* gene encoding the chloramphenicol/florfenicol efflux pump FexA [19].

MIC for linezolid and tedizolid were determined for the five isolates prior to serial passage protocol. All isolates showed linezolid susceptibility with MIC levels varying between 2 and 4 ug/ml **(**Table 1**)**. While four isolates revealed susceptibility to tedizolid, with two of them having an MIC exceeding the susceptibility cut-off (≤ 0.5 ug/ml) but still below the cut-off for intermediate resistance (1 ug/ml) [12]. The fifth isolate showed intermediate tedizolid resistance with MIC of 1.56 ug/ml **(**Table 1).

Following the induction of linezolid resistance, tedizolid MIC was determined again to check whether cross-resistance had developed. Four isolates showed a 4x increase in MIC levels with three of them turning resistant (MIC of 3 and 6 ug/ml) and one intermediate isolate (MIC of 1.56 ug/ml). However, one isolate (isolate no. 2) maintained its MIC at 0.39 ug/ml despite developing linezolid resistance and achieving growth on TSA plates with linezolid concentrations of 16 ug/ml **(**Table 2**)**.

Resultant sequencing data (two sets for each isolate; before and after developing resistance) were analyzed with the purpose of excluding mutations that may have already been occurring in our tested isolates. Mutations unrelated to our investigation were also excluded. Interpretation of the analysis results elucidates the different mechanisms of acquiring linezolid and tedizolid resistance. Mutations identified in our study can be divided into:

### Mutations conferring linezolid & tedizolid cross-resistance

Isolate no.1 showed a Single Nucleotide Polymorphism (SNP) occurring in *rpoB* gene encoding β-subunit of the DNA-directed RNA polymerase enzyme **(**Table 3**)**.

Mutations in *rpoB* gene are known to confer rifampin resistance and are commonly reported in *Mycobacterium tuberculosis* [20–22], as well as *Staphylococcus aureus* strains [23–25]. The identified SNP occurs at position 1586C > T contributing to the amino-acid change Ser529Leu. This position falls within the conserved nucleotide sequence [26] known as Rifampin Resistance Determining Region (RRDR) [27, 28] distributed over two clusters of *rpoB* gene in *S. aureus* strains [23].

The SNP identified in our study occurs at cluster II of the RRDR and was previously reported to confer rifampin resistance in *Staphylococcus aureus* strains [23] which was also the case with our isolate upon testing for acquiring rifampin resistance using disc-diffusion method. It’s important to note that the tested isolate (isolate no.1) was initially susceptible to rifampin, meaning that the acquired mutation developed as a result of the in vitro selection using linezolid. These results highly suggest the emergence of cross-resistance between rifampin and oxazolidinone antibiotics.

Although falling outside the identified RRDR, Shen et al. correlated an *rpoB* mutation in *Staphylococcus aureus* strain N315 to tedizolid resistance [17]. According to Shen and his colleagues, the detected mutation may alter regulation of the transcription process.

Although the famous 23S rRNA mutation G2576T heavily reported in the literature [7, 29] was not detected in any of the isolates, another previously reported mutation in *rplC* gene encoding the 50S ribosomal protein L3 was detected in isolate no.5 **(**Table 3**)**. This missense mutation corresponding to the amino-acid change Gly152Asp has been linked to linezolid resistance, often found coupled with other resistance mechanisms [30], or rarely found alone [7]. However, this mutation was not reported in cross-resistance to tedizolid before. This proposed cross-resistance mechanism was illustrated by the 3D structure of the ribosomes, which revealed a major part of the ribosomal protein L3 laying on the 50S ribosomal subunit’s surface, with a loop extending through the PTC [31]. L3 mutations are believed to cause conformational changes at the PTC [32], possibly altering the binding of drugs targeting this site, including linezolid and tedizolid.

### Mutations conferring linezolid resistance only

Isolate no.2 showed two missense mutations occurring in *mepB* gene part of the *mepRAB* operon contributing to the amino-acid changes Tyr2Cys and Tyr10Asn **(**Table 3**)**. The *mepRAB* operon includes three genes. *mepR*, *mepA*, and *mepB* and have been linked to antimicrobial resistance [33, 34]. *mepA* gene encodes the MepA efflux pump belonging to the MATE family transporters. MepA efflux activity extends through a variety of substrates including antibiotics, antiseptics, and disinfectants [33, 35]. Previous research had identified MepR as a regulatory repressor, repressing the expression of *mepA* gene, as well as its own [35]. Homology modeling revealed structural similarity of MepB to endonucleases [36], but its function remains unsolved.

Mutations in *mepA* and *mepR* genes have been reported and linked to changes in efflux activity in case of *mepA* mutations [34, 37], or to the overexpression of MepA efflux pump [38, 39] and the development of antimicrobial resistance in case of *mepR* mutations [34].

Isolate no.2 successfully developed linezolid resistance but maintained its tedizolid MIC levels constant without changes **(**Table 2**)**. In the absence of other linezolid resistance mechanisms, these detected mutations highly suggest the involvement of MepB in the regulation of MepA efflux pump. Our findings support a hypothesis suggesting the existence of another MepR-independent regulation system for *mepA* expression in strains overexpressing MepA without having *mepR* mutations [38]. They also come consistent with previous nucleic acid binding assays revealing MepB’s ability to bind DNA as well as RNA [36]. The increase in efflux activity of MepA towards linezolid in contrast to tedizolid could be attributed to the higher hydrophilicity and bulky structure of tedizolid compared to linezolid, whose susceptibility is known to be altered by the activity of multiple efflux pumps [40].

Although isolates no.3 and 4 successfully developed cross-resistance to both linezolid and tedizolid, a relevant mechanism that could be regarded as responsible for the acquired resistance was not concluded. This suggests the need for implementing new analysis approaches in future studies.

## Conclusion

Our study was conducted to investigate whether a mutation-mediated mechanism could confer cross-resistance to linezolid and tedizolid oxazolidinones, with further inspection of the identified mutations. In addition, the relevance of *rpoB* gene mutations to acquiring tedizolid resistance was also investigated.

In consistency with previous research, we report that indeed *rpoB* mutations are involved in acquiring tedizolid resistance. Additionally, we report the emergence of linezolid-tedizolid-rifampin cross-resistance due to a single SNP in the rifampin resistance determining region. As this mechanism is not fully understood, we recommend the future investigation of how *rpoB* mutations could potentially alter the binding sites for drugs targeting the peptidyl transferase center.

Another detected mechanism is the previously reported amino-acid change Gly152Asp of the 50S ribosomal protein L3. Although linked in multiple studies to linezolid resistance, it has never been reported before to confer resistance to both linezolid and tedizolid oxazolidinones.

Lastly, we suggest that the identified *mepB* mutations in our study affect a second regulatory mechanism of the *mepRAB* operon. This mechanism is proposed to be mediated by MepB binding to sequences falling outside the identified *mepRAB* operon.

## Electronic supplementary material

Below is the link to the electronic supplementary material.

Supplementary Material 1

## Data Availability

Sequence data generated from this study are available upon publication through the NCBI Sequence Read Archive (PRJNA1173977).
