# Supplementary material for: Identification of genetic mutations conferring tedizolid resistance in MRSA mutants
Source: Eur J Clin Microbiol Infect Dis. 2025 May 13;44(8):1917–24. doi: 10.1007/s10096-025-05157-x (PMC12321651; doi:10.1007/s10096-025-05157-x)
Supplement: Supplementary file 1 — Supplementary Material 1 [file 10096_2025_5157_MOESM1_ESM.pdf]

European Journal of Clinical Microbiology & Infectious Diseases

Identification of genetic mutations conferring tedizolid resistance in MRSA mutants

Nesma B. Goda[1 *], Amira M. El-Ganiny[2], Tharwat R. El-khamissy[1], Fares Z. Najar[3], Ashraf A. Kadry[2]

* Email: nesma-barbary@eru.edu.eg
ORCID ID: https://orcid.org/0000-0001-6075-155X

[1]Microbiology and Immunology Department, Faculty of Pharmacy, Egyptian Russian University, Badr, Egypt

[2]Microbiology and Immunology Department, Faculty of Pharmacy, Zagazig University, 44519, Zagazig, Egypt

[3]High-performance Computing Center MS #105 Oklahoma State University, Stillwater, OK 74078, USA

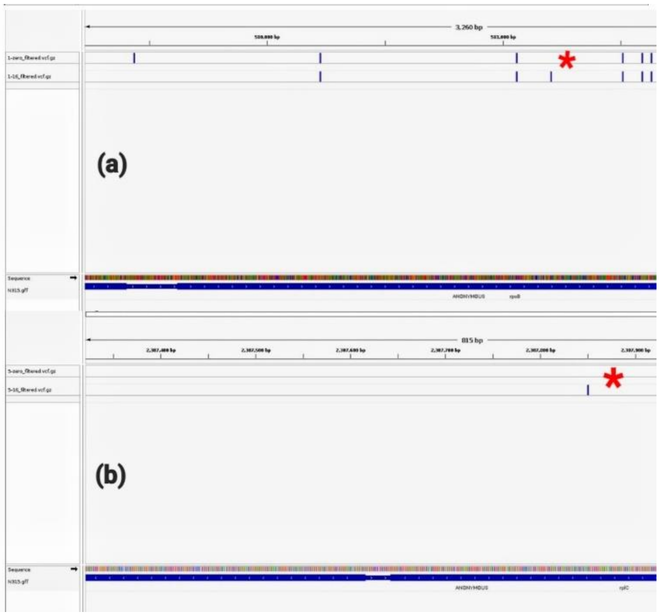

Fig 1 Mutations conferring linezolid & tedizolid cross-resistance visualized by Integrative Genomics Viewer (IGV). (a) The star indicates a unique *rpoB* gene variant occurring in mutant no. 1 (1-16) following the development of cross-resistance, compared to isolate no. 1 (1-zero) before the induction of resistance.

(b) The star indicates a unique *rplC* gene variant occurring in mutant no. 5 (5-16) following the development of cross-resistance, compared to isolate no. 5 (5-zero) before the induction of resistance

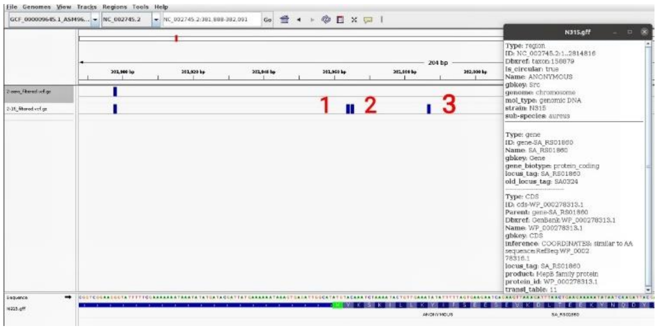

Fig 2 Mutations conferring linezolid resistance only visualized by Integrative Genomics Viewer (IGV). 1, 2, and 3 refer to unique *mepB* variants occurring in mutant no. 2 (2-16) following the development of linezolid resistance, compared to isolate no. 2 (2-zero) before the induction of resistance.

1 and 3 are missense variants, 2 is a synonymous variant and therefore was not reported as a contributor to the acquired resistance. **SA_RS01860** is the symbol for *mepB* gene within the SnpEff database for *S. aureus* N315.
